# Janus kinase inhibitors are of limited use in refractory JIA-associated uveitis: retrospective data from a tertiary uveitis center

**DOI:** 10.1186/s12969-026-01243-2

**Published:** 2026-07-11

**Authors:** Karoline Baquet-Walscheid, Charlotte Wortmann, Kai Rothaus, Charlotte Ohlmeier, Carsten Heinz, Daniel Windschall, Arnd Heiligenhaus

**Affiliations:** 1 MVZ for Laboratory Medicine and Microbiology Koblenz-Mittelrhein, Viktoriastraße 35-39, 56068 Koblenz, Germany; 2 Department of Ophthalmology, University of Duisburg-Essen, Essen, Germany; 3 Department of Ophthalmology, St. Franziskus Hospital, Muenster, Germany; 4 Pediatric Rheumatology, St. Josef-Stift, Sendenhorst, Germany; 5 Faculty of Medicine, Martin Luther University of Halle Wittenberg, Halle, Germany

**Keywords:** Juvenile idiopathic arthritis, Uveitis, DMARDs, JAK inhibitors, Paediatric/juvenile/child/adolescent, Retrospective study

## Abstract

**Background:**

Juvenile idiopathic arthritis-associated uveitis (JIAU) frequently follows a chronic and complicated course. Janus kinase inhibitors (JAKi) represent a promising new therapeutic option.

**Methods:**

Retrospective monocentric analysis of 20 children with chronic anterior JIAU treated with tofacitinib (*n* = 18), baricitinib (*n* = 1) or upadacitinib (*n* = 1). Outcome measures: uveitis or arthritis inactivity at any time during follow-up, recurrences after achieving inactivity, sparing of medication, occurrence of new ocular complications, resolution of preexisting macular edema.

**Results:**

All patients had received methotrexate prior to baseline, and at least two biologics had been ineffective in 18/20 patients. Whilst receiving JAKi medication, uveitis was inactive in eight patients (anterior chamber cell count < 0.5+) at any time during follow-up (mean 9.2 ± 3.3 months), but uveitis relapsed in 6 of them subsequently. In 7 patients, treatment was terminated after ≤ 1 year due to inadequate response of ocular inflammation. During follow-up, new uveitis-related complications occurred in 7 patients (9 eyes), the most frequent were macular edema (8 eyes of 7 patients), and ocular hypertension (5 eyes of 3 patients). Reduction of concomitant systemic medication was possible in 3/14 patients. Any reduction of topical steroids during follow-up was possible in 24/30 eyes, and 23/30 eyes received a lower dose at the last follow-up than they did at baseline.

**Conclusions:**

Our data show limited effectiveness of JAKi treatment in patients with chronic DMARD-refractory JIAU. The number of patients in whom uveitis quiescence could be observed was low, as was the possibility for reduction of concomitant medication, and the relapse rate was high.

**Clinical trial number:**

Not applicable.

## Background

Juvenile idiopathic arthritis (JIA) is the most common rheumatological disease in childhood and adolescence. A significant number of children (about 11–22%) develops associated uveitis (JIAU), which mainly occurs bilaterally and leads a chronic course [1, 2]. The inflammation of both joint or eye can lead to various complications affecting function and quality of life. To reduce complications and improve long-term prognosis, consistent anti-inflammatory therapy of both joint and eye inflammation is necessary. According to the consensus of the current international guidelines on JIAU [3–7], topical steroids are initially used for the treatment of uveitis. If ocular inflammation persists, complications develop, or high topical steroid dosages are required over time, systemic therapy with disease modifying antirheumatic drugs (DMARDs) is indicated. In general, the treatment strategy follows a step-up approach [8]: Initially, most patients receive methotrexate (MTX). In case of incomplete response or severe side effects, a TNF inhibitor is added, with adalimumab presently being preferred as the only biologic approved for JIAU. However, there is a significant number of children and adolescents, in whom sustained quiescence of ocular inflammation cannot be achieved by a TNF inhibitor, either given alone or combined with MTX [7, 9]. Various other biological DMARD options, such as anti-IL-6 (tocilizumab), selective costimulatory modulators (abatacept), or anti-CD20 (e.g., rituximab) are available, but none of those has been approved for the use in JIAU.

Janus kinase inhibitors (JAKi) represent a new therapeutic option for JIA [10–12]. Janus kinases (JAKs) are intracellular enzymes with a variety of functions in immunological and inflammatory processes. The “JAK family” includes four isoforms (JAK1, JAK2, JAK3, and Tyk2 [tyrosine kinase 2]), which only become active after di- or trimerization and phosphorylation. In short, the binding of proinflammatory cytokines to cell receptors initiates an intracellular signaling cascade with di- or trimerization and phosphorylation of the respective JAK isoforms. These can then bind so-called “STAT” proteins (signal transducer and activator of transcription), which, after dimerization, stimulate the transcription of various factors (including proinflammatory cytokines, growth factors, and others). Depending on the preparation, JAKi are able to bind different JAKs and thereby block phosphorylation, leading to inhibition and thus interruption of the intracellular signaling cascade. The effect and side effect profile differ depending on the JAK isoform targeted. JAKi have been used for several years in various autoimmune diseases, such as rheumatoid arthritis or chronic inflammatory bowel diseases. In Germany, tofacitinib and baricitinib received approval for several JIA subgroups, of which the extended oligoarticular and RF-negative polyarticular subgroup confer an especially high risk for development of uveitis [1, 4]. However, JAKi agents have not been approved for the use in JIAU yet. The phase 3 *Juve-Bright* trial (NCT04088409) directly compared baricitinib (*n* = 24) vs. adalimumab (*n* = 5) for JIA-associated and ANA-positive chronic anterior uveitis [13]. In contrast to several promising case reports reporting good efficacy of JAKi preparations in JIAU [9, 14–16], the primary endpoint was not met in this trial, as only about one third of patients responded properly to treatment [13].

Due to the currently limited treatment options approved for JIAU and the frequent therapy-refractory courses, further insights into the effectiveness of JAKi in JIAU would be desirable. The current study aims to provide data on the course of JIAU under JAKi therapy.

## Methods

The effectiveness of JAKi treatment in patients with JIAU was evaluated in a monocentric retrospective cohort study of patients with JIAU diagnosed between 2002 and 2020. Patients were eligible for study inclusion if (a) they had been diagnosed with JIA-associated insidious onset anterior uveitis in accordance with both ILAR (International League of Associations for Rheumatology [17] and SUN (Standardization of Uveitis Nomenclature criteria [18]), were (b) started on JAKi treatment due to uveitis refractory to prior conventional synthetic (cs)- and biologic (b)DMARD treatment, and were (c) younger than 18 years of age at the time of treatment initiation. As this was only a retrospective evaluation, there was no number / type of DMARDs a patient had to fail, but initiation of JAKi treatment was based on the uveitis specialist’s assessment of the prior clinical course of disease. Uveitis activity at baseline (the visit at which JAKi treatment was initiated) was no prerequisite for study inclusion, as there might often have been a delay between the decision to switch to JAKi treatment and the initiation of the respective medication.

The study was conducted in accordance with the Declaration of Helsinki and was approved by the local ethics committee (Ethikkommission der Ärztekammer Westfalen-Lippe und der Universität Münster; reference number 2021-517-f-S). Due to the retrospective nature of the study, no written informed consent needed to be obtained from patients or parents.

SUN criteria [18] were used to diagnose and classify uveitis and grade activity. An AC (anterior chamber) cell grade of ≥ 0.5 + AC cells was considered as active uveitis, whereas an AC cell grade of < 0.5 + was judged as inactive uveitis [18]. The following clinical parameters were documented at each visit, and were documented via a standardized case report form: Visual acuity (in logarithm of the minimum angle of resolution [logMAR]), intraocular pressure (IOP), results of slit-lamp examination (including assessment of uveitis activity), dilated fundoscopy and OCT imaging as well as laser-flare measurements (LFM), if performed. Any uveitis-related complications were documented (cataract, glaucoma and ocular hypertension, ocular hypotony, posterior synechiae, macular edema, optic disk edema, epiretinal membrane, retinal detachment, uveitis-induced amblyopia). Any systemic and uveitis-related topical or local (i.e., corticosteroid injections) medication prior to the baseline visit and at each follow-up visit was documented.

In all patients, JAKi treatment was initiated for either active uveitis and/or arthritis after exclusion of contraindications by a pediatric rheumatologist, and monitored regularly by assessment of clinical and laboratory parameters. All treatment decisions regarding systemic DMARD use were based on interdisciplinary networking between uveitis specialist and pediatric rheumatologist.

The following visits were analyzed: Prior to start of JAKi treatment (baseline; BL), and at 3, 6, 9, and 12 months after initiation. In unilateral uveitis, only the diseased eye was analyzed. In bilateral uveitis, the eye with the higher anterior chamber cell grade at baseline was selected as the study eye. If both eyes had identical AC cell scores at BL, the right eye was assigned to be the study eye.

The primary outcome measure was defined as uveitis inactivity at any time during follow up, defined as an AC cell grade of < 0.5 + in both eyes. Secondary outcomes were (a) recurrences after documented uveitis inactivity, (b) achievement of arthritis inactivity, (c) sparing of topical or concomitant systemic corticosteroids, and/or DMARD, (d) occurrence of new ocular complications, and (e) resolution of preexisting macular edema. When assessing the reduction of concomitant systemic medication, the definition by Saurenmann et al. [19] was applied, i.e., good response was defined as ≥ 50% decrease in both corticosteroid use and DMARD agent; moderate response was defined as ≥ 50% decrease in either corticosteroid or DMARD agent; and poor response was defined as < 50% decrease in both corticosteroid and DMARD agent [19]. For this analysis, only patients with csDMARD use at BL were taken into consideration, as all bDMARDs were terminated at the start of JAKi treatment.

The results are presented using descriptive statistics. For categorial data, the absolute and relative frequencies are presented, whereas for numerical data median and interquartile range (IQR) or mean and standard deviation are reported.

## Results

### Patient characteristics at baseline visit

Patients started JAKI therapy at a median age of 17.0 years (IQR 4.44 years) and displayed characteristics typical for JIAU: early onset of both arthritis and uveitis as well as predominance of the female sex (75%), oligoarticular JIA subtype (55%) and ANA positivity (100%). All patients had been diagnosed with chronic, asymptomatic anterior uveitis (bilateral in 19/20 patients). Uveitis complications were present in 38/40 eyes (see Table 1), and ocular surgery had been performed in 4 patients (5 eyes) prior to BL. Only two patients had macular edema (ME) at baseline. At BL, uveitis was active in 16 patients, with a median LFM of 36.8 (IQR 77.1) ph/ms), and median BCVA (best-corrected visual acuity) of 0.2 (IQR 0.4) logMAR per study eye. An AC cell score of zero was observed in 15 (38.5%) eyes, whereas 11 eyes (28.2%) presented with an AC cell score of 0.5, and 12 eyes (30.8%) had ≥ 1 + AC cells. Arthritis was active at BL in 4 patients. All patients had received MTX therapy prior to BL, and adalimumab had been given to almost every patient as well (19/20).

**Table 1:** Patient characteristics at baseline visit

**Age at baseline** [years, median (IQR)]	17.0 (4.44)
**Female sex** [n (%)]	15 (75)
**Age at JIA diagnosis** [years, median (IQR)]	2.0 (2,81)
**Age at uveitis diagnosis** [years, median (IQR)]	3.67 (4.71)
**ILAR classification** [n (%)]	
Persistent oligoarthritis	3 (15)
Extended oligoarthritis	8 (40)
Polyarthritis RF negative	8 (40)
Polyarthritis RF positive	1 (5)
**ANA pos.** [n (%)]	20 (100)
**HLA B27 pos.** [n (%)]	3 (15)
**RF pos.** [n (%)]	1 (5)
**Active arthritis** [n (%)	4 (20)
**DMARD treatment at baseline** [n (%)]	
csDMARD	13 (65)
MTX	12 (60)
bDMARD	10 (50)
Adalimumab	1 (5)
other TNFalpha inhibitors	5 (25)
Tocilizumab	3 (15)
**DMARD treatment (any previous);** [n (%)]	
csDMARD	20 (100)
MTX	20 (100)
bDMARD	19 (95)
Adalimumab	18 (90)
other TNFalpha inhibitors	14 (70)
Tocilizumab	12 (60)
**Uveitis bilateral** [n (%)]	19 (95)
**Previous ocular surgery** [n, eyes/patients]	5 / 4
Cataract surgery	3 / 3
Any other surgery	4 / 4
subtenon / intraocular injection	2 / 2
AC cell score*	
0 [n (%)]	15 (38.5)
0.5 [n (%)]	11 (28.2)
1+ [n (%)]	8 (20.5)
≥ 2+ [n (%)]	4 (10.3)
**LFM** [photons /ms; median (IQR)]*	36.8 (77.1)
**BCVA** [logMAR; median (IQR)]*	0.2 (0.4)
**Ocular complications** [number of eyes]*	
any	38
Cataract	23
Band keratopathy	7
Posterior synechiae	16
Ocular hypertension	2
Glaucoma	8
Macular edema	2
Epiretinal membrane	5
Optic disc edema	4
Ocular hypotony	0
Retinal detachment	0
Amblyopia	0

IQR interquartile range; JIA juvenile idiopathic arthritis; ILAR international league of associations for rheumatology; RF rheumatoid factor; ANA antinuclear antibody; DMARD disease modifying antirheumatic drug; cs conventional synthetic; b biological; AC anterior chamber; LFM laserflare measurement; ms millisecond; BCVA best-corrected visual acuity; logMAR logarithm of the minimum angle of resolution; * per study eye at baseline

AC cell score was reported in accordance with the SUN classification (Jabs et al. 2005)

Data from individual patients are displayed in Table 2, showing that most patients had received numerous DMARD preparations prior to initiation of JAKi treatment, and the frequency of topical corticosteroid drops was high in the majority of patients. In 4 patients, all previously given systemic DMARD therapy had been stopped prior to or at BL due to ineffectiveness and / or side effects.

**Table 2:**
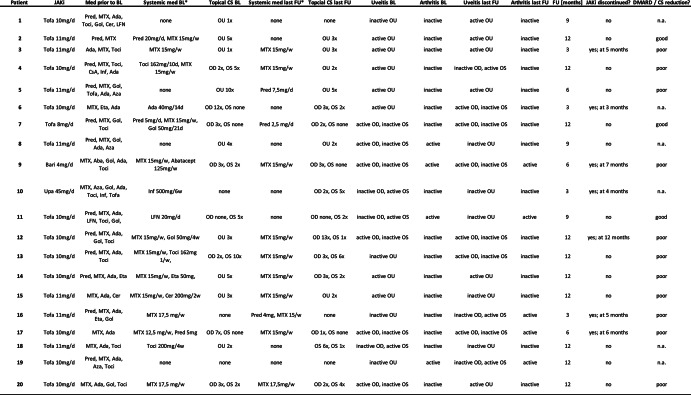
Characteristics and course of disease in individual patients

JAKi=janus kinase inhibitor; med=medication; BL=baseline; CS=corticosteroids (drops per day; equivalent of prednisolone acetate 1% formulation); FU=follow-up; Tofa=tofacitinib; d = day; Pred=prednisolone; Ada=adalimumab; Toci=tocilizumab; Gol=golimumab; Cer=certolizumab; LFN=leflunomide; OD=right eye; OS=left eye; OU=both eyes; Inf=infliximab; CsA=ciclosporine A; Aza=azathioprine; Eta=etanercept; Aba=abatacept; Bari=baricitinib; Upa=upadacitinib; n.a.=not applicable

* in addition to JAKi preparation

### Follow-up under treatment

The mean duration of follow-up was 9.2 ± 3.3 months. A total number of 8 patients had bilateral uveitis inactivity at any time during follow-up, after a mean duration of 6.4 (± 2.9) months of JAKi treatment. However, uveitis relapsed in 6 of these 8 patients at some point during the subsequent follow-up (Table 3).

**Table 3:** Course of disease under JAKi treatment

**Disease activity**	
**Active uveitis at BL** [n; patients]	16
**Active arthritis at BL** [n; patients]	4
**Ocular quiescence at any time during FU** [n; patients]	8
**Mean duration until uveitis quiescence** [months ± SD]	6.4 ± 2.9
**Recurrence after achieving quiescence** [n; patients]	6
**Arthritis inactivity at any time** [n; patients]	2
**Mean duration until arthritis inactivity** [months ± SD]	3.0 ± 0
**Sparing of systemic corticosteroids and/or DMARDs** [n; patients]*	
Good	3
Moderate	0
Poor	11
**Topical corticosteroid use** [n; eyes]	
Any, at BL (total number of uveitis-affected eyes: *n* = 39)	30
Reduction at any time during FU	24
Reduction to ≤ 2 drops daily	14
Reduction at final visit compared to BL	23
**Patients with new complications during FU** [n; eyes/patients]	
Cataract	2 / 2
Band keratopathy	1 / 1
Posterior synechiae	1 / 1
Ocular hypertension	5 / 3
Glaucoma	3 / 2
Macular edema	8 / 7

BL baseline; FU follow-up; SD standard deviation

* definition: see material & methods section; according to Saurenmann RK, Levin AV, Rose JB, et al. Tumour necrosis factor alpha inhibitors in the treatment of childhood uveitis. Rheumatology (Oxford) 2006;45:982-9

Response to treatment regarding arthritis activity was observed in 2/4 patients with active arthritis at baseline at any time during follow-up, with a mean duration of 3 (± 0) months until achieving inactivity. One of the patients responded regarding arthritis activity, but did not show any response regarding uveitis activity.

In seven patients, JAKi treatment was terminated due to inadequate response of ocular inflammation after a mean duration of 6.0 (± 2.9) months (Tables 2 and 3). Three of those were also active regarding arthritis disease (two of them had already had active arthritis at BL, whereas one had been inactive at BL, but relapsed under JAKi treatment). None of the patients reported any subjective adverse effects under JAKi therapy, and no patient needed to discontinue JAKi treatment due to objectively documented side effects.

When assessing the potential reduction of systemic medication (not applicable for 6 patients: 3 without systemic medication at BL, and 3 with bDMARDs only, which were terminated directly at BL), we found that only three patients showed good response as defined above. However, only one of those three patients had inactive uveitis at the final follow-up; in the remaining two patients, systemic medication had been reduced during the course of disease, but uveitis relapsed until the final follow-up. DMARD sparing was poor in the remaining 11 patients. Regarding the use of topical steroids, any reduction during follow-up was possible in 24/30 eyes, and 23/30 eyes received a lower dose at the last follow-up than they did at study inclusion. However, the desired reduction to two drops or less per eye per day was achieved in only 14 eyes at the latest follow-up visit.

During follow-up, new complications occurred in 7 patients (9 eyes), the most frequent complications being macular edema (8 eyes of 7 patients) and ocular hypertension (5 eyes of 3 patients), leading to a total number of 10 eyes with ME (two prior to initiation of therapy and 8 with new onset ME under JAKi treatment). In two eyes (one with preexisting and one with new onset ME), macular edema resolved under continued JAKi treatment. Regarding the remaining 8 eyes, macular edema improved during continuation of JAKi intake in 3 of them (one with preexisting and two with new onset ME), whereas the therapy needed to be discontinued in the remaining 4 individuals (5 eyes) due to treatment failure.

## Discussion

Our data disclose limited effectiveness of JAKi treatment in JIA patients with long-standing course of uveitis refractory to multiple DMARD regimens. The number of patients in whom uveitis quiescence could be observed was low, as was the sparing of both concomitant systemic medication and topical steroids, while the relapse rate during the course of treatment was high.

There is currently little data on the efficacy of JAK inhibitors in JIA-associated uveitis (JIAU). Initial data, resulting from case reports, seemed promising: A retrospective case series with three children under baricitinib and one child under tofacitinib therapy showed improvement in uveitis findings in all patients [16]. A case report of an adult patient with JIAU [15] demonstrated good efficacy of the treatment on both uveitis activity and accompanying macular edema, a common vision-threatening complication of JIAU. The largest case series published [20] yet encompasses retrospective data from nine children with uveitis (half of them diagnosed with anterior uveitis) treated with tofacitinib. The authors report remission without topical steroids in 6 patients, and relapse of anterior uveitis in only two children at the end of the follow-up (median 277 days) [20].

Given these prior publications, the limited effectiveness we now observe in our present cohort seems disappointing. However, our current findings are in line with the only prospective data on JAKi (namely, baricitinib) in JIAU. These results come from a phase 3 trial (Juve-Bright) analyzing data from 24 children with active JIAU or chronic anterior ANA-positive uveitis who had an inadequate response to MTX (*n* = 5) or to both MTX and bDMARDs (*n* = 19) [13]. Those were compared to *n* = 5 patients receiving adalimumab after failing MTX. Here, the primary efficacy endpoint was the proportion of responders at week 24 (w24), defined according to the SUN criteria as a 2-step decrease in the level of inflammation (AC cells) or decrease to zero through w24 in the eye most severely affected at baseline [13]. In the baricitinib group, only 8 (33%) of patients achieved a response at w24 (7 with prior bDMARD and MTX treatment, and one with prior MTX treatment only), compared to 4 (80%) in the adalimumab group [13].

However, one has to keep in mind that these response rates are not directly comparable to our population, as all (except one) of our patients had been non-responders to not only MTX, but a minimum of one (frequently several) bDMARDs as well as MTX as a combination therapy. As documented by Table 2, the majority of our patients received JAKi treatment as a fourth- or even fifth-line therapy in the course of a chronic, highly inflammatory process going on for many years. In addition, not only the study populations but also the selected endpoints and the treatment regimens (JAKi agent) are not directly comparable: Whereas patients in the prospective Juve-Bright study cohort were treated with Baricitinib, the majority of patients in our cohort received Tofacitinib.

Given the well-known differences in the effects of various JAK inhibitors on the different JAK isoforms, a variable biological effect would theoretically be conceivable. However, the currently published literature does not provide substantial support for such an effect. Nevertheless, there is a case report of an adult patient with JIAU in whom treatment with Upadacitinib was initiated due to secondary treatment failure under Tofacitinib, resulting in at least a temporary marked clinical improvement [14].

Another factor to consider is the dosage of the agents: In our current cohort, 9/18 patients received Tofacitinib at a dosage of 10 mg/day, whereas 8/18 patients received 11 mg/day as an extended-release formulation. The cohort is too small (and the age and weight range too heterogeneous) to assess an effect of the administered dose, but it is conceivable that—similar to what has partly been observed with TNF inhibitors—higher dosages than those typically used for arthritis may be required.

It might be speculated that response to treatment of uveitis with JAKi might be better, if the medication were initiated during an earlier stage of this chronic disease, which has been observed previously for other DMARD preparations. Nevertheless, even in our cohort of heavily pretreated patients with a long-standing, chronic disease course, a partial success in terms of achieving inflammatory remission was often observed. Although relapses frequently occur, meaning that the duration of the effect is limited, a certain therapeutic benefit can nevertheless be identified.

Limitations to our data result from the number of patients we were able to document. Due to the rarity of the disease and the fact that JAKI can currently only be used in off-label situations for uveitis, or be prescribed due to arthritis activity, the number of patients we were able to document is correspondingly limited. As our analysis was retrospective, we cannot delineate (confounding) effects of any concomitant topical or systemic medication, and duration of follow-up varied.

According to our data and the current evidence from the literature, JAK inhibitors (tofacitinib, baricitinib, upadacitinib) may be an option for treatment of refractory JIAU, with evidence of disease control and steroid-sparing effects in some patients. Tolerability and safety regarding side effects seems to be favorable. As both MTX and adalimumab as the currently recommended first and second line treatment options are effective in a high number of patients, JAKi preparations will probably not be considered for RCTs analyzing their use as a first-line therapy. However, given the fact that oral administration is a huge advantage of JAKi, especially in the pediatric population, a non-inferiority trial could be a relevant approach. Further larger studies are needed to define their role in the step-up treatment approach in JIAU.

## Conclusions

In pediatric patients with uveitis refractory to MTX and multiple bDMARs, JAK inhibitors can be of use as an individual treatment attempt. Our retrospective data from a cohort of 20 patients show limited effectiveness in chronic DMARD-refractory JIA uveitis. Uveitis inactivity can be observed, but is often transient and requires additional medication.

## Data Availability

The datasets used and/or analysed during the current study are available from the corresponding author on reasonable request.
